# Assessment of Foliar Uptake and Accumulation of Airborne Polyaromatic Hydrocarbons Under Laboratory Conditions

**DOI:** 10.1007/s00128-020-02814-z

**Published:** 2020-03-09

**Authors:** Gábor Teke, Katalin Hubai, Dorina Diósi, Nora Kováts

**Affiliations:** 1 ELGOSCAR-2000 Environmental Technology and Water Management Ltd., Balatonfuzfo, 8184 Hungary; 2 Institute of Environmental Sciences, University of Pannonia, Egyetem str. 10, Veszprém, 8200 Hungary

**Keywords:** Air pollution, Polyaromatic hydrocarbons, Urban gardening, Vegetative vigour test, Bioaccumulation, Bioconcentration factor

## Abstract

Urban horticulture and community gardening have become more and more popular in the past years, however, the risk of bioaccumulation of atmospheric polyaromatic hydrocarbons (PAHs) in vegetables grown in polluted areas cannot be neglected. In our study, the No. 227 OECD GUIDELINE FOR THE TESTING OF CHEMICALS: Terrestrial Plant Test: Vegetative Vigour Test was followed to assess foliar uptake of PAHs from aqueous extract of an urban aerosol. Using lettuce (*Lactuca sativa*) as a test organism, significant accumulation was experienced. The highest bioconcentration factors (BCFs) were experienced for naphthalene and for anthracene, pyrene and fluoranthene showed the lowest bioaccumulation potential. BCF of each PAH showed strong correlation with molecular weight. The standard protocol defined by the Guideline made it possible to assess bioaccumulation pattern under controlled laboratory conditions.

The popularity of urban horticulture has increased considerably in the past years, with the motifs of growing healthy vegetables from controlled sources and of community building. However, the risk posed by kitchen vegetables cultivated in urban areas cannot be neglected (Uzu et al. 2014).

There are early studies available reporting the risk of bioaccumulation of atmospheric polyaromatic hydrocarbons (PAHs) in vegetables grown in industrial areas (Voutsa et al. 1996; Kipopoulou et al. 1999). Atmospheric deposition resulted in significant accumulation of 16 priority PAHs in vegetables grown in the vicinity of thermal power plants (Khillare et al. 2012). Uptake of 15 PAHs from the atmosphere was reported by Xiong et al. (2017) in cabbage. Arable crops might also be affected in polluted areas (Tian et al. 2018). PAH deposition on grasslands might even pose hazard to grazing dairy ruminants (e.g. Dan-Badjo et al. 2007).

PAHs are generated during the incomplete combustion of organic materials such as fossil fuel-burning in motor vehicles or during residential heating, etc. In the air, most of the PAHs with low vapour pressure are adsorbed on particles. Many PAHs are considered highly carcinogenic or mutagenic; for example the so-called Car-PAHs, namely benzo(a)anthracene, chrysene, benzo(b)fluoranthene, benzo(k)fluoranthene, benzo(a)pyrene, dibenzo(a,h)anthracene, indeno(1,2,3-cd)pyrene and benzo(g,h,i)perylene (reviewed by Srogi 2007). The US Environmental Protection Agency (EPA) enlists 16 priority PAHs (EPA–PAHs) posing the highest environmental risk (reviewed by Keith 2015).

Dietary uptake is considered the major exposure route of PAHs (Ramesh et al. 2004). Due to the high lipophilicity, PAHs can be readily absorbed from the gastrointestinal tract and accumulate in various tissues (Abdel-Shafy and Mansour 2016). It has been shown that they might hinder the metabolic activity of glucose homeostasis (Bansal and Kim 2015).

While field bioaccumulation studies might provide useful data on actual risk posed by consuming polluted vegetables (e.g. Pandey et al. 2012; Wang et al. 2017), they lack basic information such as spatial or temporal distribution of contaminants or duration of exposure. Doucette et al. (2018) reviewed over 350 papers dealing with bioaccumulation studies and concluded that the diversity of experimental approaches and lack of basic data such as exposure or plant growth conditions make inter-study comparisons and bioconcentration estimations extremely difficult. The authors suggested that standard test protocols should be introduced.

As such, our main goal was investigate if a standard protocol, the No. 227 OECD GUIDELINE FOR THE TESTING OF CHEMICALS: Terrestrial Plant Test: Vegetative Vigour Test, can be adopted for bioaccumulation studies. The Guideline was originally introduced in herbicide regulation to test general chemicals, biocides and crop protection products (Boutin et al. 2012), but with some modifications, it has proven suitable to test the deleterious effects of the water-soluble components of airborne particulate matter (Kováts et al. 2017).

Present study was targeted to assess the accumulation of PAHs from aerosol, establishing stressor–effect relationships.

## Materials and Methods

PM2.5 aerosol samples were collected in Budapest (Hungary), between 02.12.2016 and 28.12.2016. The sampling site is maintained by the Hungarian Meteorological Service and is located in a suburban area of the city. A high volume sampler Digitel (DHA-80) was employed, samples were collected on glass fibre filters (Whatman QMA, diameter 150 mm). The sampling time was 24 h. Filters were stored in a freezer at − 20°C until use.

In order to gain extract of sufficient volume, a single composite sample was produced, by using the halves of each filter. For extraction of the water soluble compounds, they were cut in pieces and placed in beaker filled with 1000 mL high purity (MilliQ) water. The filter pieces were stirred in water several times, then the beaker was covered and the extraction continued for 24 h at room temperature. Finally, the extract was filtered on 0.45 µm pore size filter (Varga et al. 2001).

Lettuce (*Lactuca sativa*) was selected as a test organism. This species is not only recommended by the Guideline, but it has been a very frequented test organism due to high foliar surface (e.g. Schreck et al. 2012, 2013) and also being a very popular vegetable in kitchen gardens. In comparative studies, its bioaccumulation capacity is generally higher than that of other vegetables (e.g. Li et al. 2015). Phytotoxicity testing was performed based on the protocol given in the Guideline. Shortly, plants were grown in pots of 15 cm diameter in commercial soil [(pH 6.8 ± 0.5; N (m/m%): min 0.3; P_2_O_5_ (m/m%): min 0.1; K_2_O (m/m%): min 0.3)]. Testing was conducted in a glass-house, environmental conditions were in concordance with the prescriptions of the Guideline (temperature: 22 ± 10°C; humidity: 70% ± 25%; photoperiod: minimum 16 h light; light intensity: 350 ± 50 µE/m^2^/s).

Treatment started when the plants reached the 4-true leaf stage. 30 plants were used in the test group and 30 in the control group. Test plants were sprayed with the aqueous extract on Day1, Day 8, and Day 15. Control plants received spraying with tap water. The test was terminated on Day 22.

Analytical determinations were performed in the testing laboratory at the Laboratory of the ELGOSCAR-2000 Environmental Technology and Water Management Ltd. accredited by the National Accreditation Authority, registration number NAH-1-1278/2015.

PAH concentrations in the aerosol extract were measured according to MSZ 1484-6:^2003^ standard [(MSZ 1484-6:^2003^: Testing of waters. Determination of polycyclic aromatic hydrocarbons (PAH) content by gas chromatographic–mass spectrometry, LOD: ng/L)]. The plant samples were analysed by Agilent 6890GC 5973E MSD GC–MS based on MSZ EN 15527:^2009^ (Characterization of waste). Determination of polycyclic aromatic hydrocarbons (PAH) in waste using gas chromatography mass spectrometry (GC/MS, LOD: 0.1 µg/kg).

In order to achieve a homogeneous and representative plant sample 10 g of lettuce leaves was grinded and pounded with 10 g anhydrous sodium sulphate in a ceramic mortar. 10 g of the samples was extracted three times with Ultrasonic extraction for 20 min with 20 mL n-hexane. Prior to extraction 10 mL acetone was added and the samples were spiked with 100 µL of 0.01 µg/mL deuterated PAH surrogate mixture containing Naphtalene-d8, Acenaphthene-d10, Phenanthrene-d10, Chrysene-d12, Benzo(a)pyrene-d12, and Perylene-d12 (Restek Corporation, USA). After the extraction the sample extract was concentrated in a dry nitrogen stream to 1 mL. With each sample an additional solid phase silica gel and alumina oxide sample clean-up was performed. For GC–MS measurements, an HP-6890 gas chromatograph was coupled to an HP-5973 quadrupole mass spectrometer (low-resolution single MS) (Agilent Technologies, Palo-Alto, USA).

## Results and Discussion

Altogether 19 PAHs were found in the extract, including the 16 priority PAHs enlisted by US EPA. With the exception of dibenzo(a,h)anthracene, these PAHs were accumulated in the leaves. Table 1 gives the composition of the PM2.5 extract and the concentration of the accumulated compounds in lettuce. Also, BCF was calculated (Kacálková and Tlustoš 2011) following the equation:

$$BCF = \frac{{{\text{PAH concentration in treated lettuce }}\left( {{\upmu {\text{g}}}/{\text{L}}} \right)}}{{{\text{PAH concentration in the PM}}2.5{\text{ sample }}\left( {{\upmu {\text{g}}}/{\text{kg}}} \right)}}$$

**Table 1:** Concentration of PAHs in the aerosol extract and in the lettuce leaves

PAH	PM2.5 sample (µg/L)	Treated lettuce (µg/kg)	BCF	Molecular weight (g/mol)
**Naphthalene**	0.39	72	184.61	128.17
2-Methyl-naphthalene	0.19	22.85	120.26	142.20
1-Methyl-naphthalene	0.15	11.55	77.00	142.20
**Acenaphthylene**	0.02	2.6	130.00	152.19
**Acenaphthene**	< LOD	1.05	< LOD	154.21
**Fluorene**	0.04	3.55	88.75	166.22
**Phenanthrene**	0.39	15.55	39.87	178.23
**Anthracene**	0.03	6.15	205.00	178.23
**Fluoranthene**	0.59	12.15	20.59	202.25
**Pyrene**	0.59	11.4	19.32	202.25
**Benzanthracene**	0.15	7.45	49.67	228.29
**Chrysene**	0.27	7.6	28.14	228.30
**Benzo(b)fluoranthene**	0.22	11.35	51.59	252.31
**Benzo(k)fluoranthene**	0.07	3.45	46.28	252.31
Benzo(e)pyrene	0.1	3.7	37.00	252.32
**Benzo(a)pyrene**	0.09	4.55	50.56	252.32
**Indeno1,2,3CD-Pyrene**	0.12	4	33.33	276.33
**Benzo(g,h,i)perylene**	0.08	2.8	35.00	276.30
**Dibenzo[a,h]anthracene**	0.01	< LOD	< LOD	278.35
Total PAH	3.53	205.5	58.22	–

Bioconcentration factors (BCFs) are also indicated. Priority PAHs are given in bold

Strikingly high BCFs were experienced for naphthalene (184.61) and for anthracene (205). The concentration of naphthalene in the aqueous PM2.5 extract was already relatively high (0.396 µg/L), much higher than in a winter urban PM10 sample (Kováts et al. 2017). In general as particulate matter size decreases, relatively more potentially toxic compounds are bound; e.g. Valavanidis et al. (2006) reported that the fine particulate PAHs concentrations were higher than coarse particles. Pyrene and fluoranthene showed the lowest BCF (19.32 and 20.59 respectively). In the control plants, no PAHs were detected.

Most studies calculate BCF for uptake and accumulation of PAHs from soil (e.g. Zohair et al. 2006; Zhang et al. 2015; Inam et al. 2016). Mo et al. (2009) for example report that highest BCF of total PAHs (5.5) was found in *Brassica* sp. collected in the Pearl River Delta (South China). Khan and Cao (2012) calculated RCFs (root/soil concentration factor) and SCFs (shoot/soil concentration factor) for different vegetables grown in metropolitan areas of Beijing (China) and found that the bioaccumulation factors decreased with the increase of ring numbers. Nevertheless, several studies have shown that the major pathway for the accumulation of PAHs in vegetation is atmospheric deposition (Jia et al. 2019). Li et al. (2008) for example did not find correlation between total PAHs in vegetable samples with soil samples. Some data are available, however, on the relationship between atmospheric concentration of PAHs and their accumulation in higher plants. In the study of Sharma and Tripathi (2009), BCFs of total PAHs in the leaves of *Calotropis gigantea* (an evergreen shrub) were in the range of 1.00–11.72, while BCF for total PAHs was 58.22 in our experiment. Different species are, however, difficult to compare due to differences in important attributes such as leaf morphology or life cycle (Franzaring and van der Eerden 2000).

Studies reporting PAH accumulation specifically in lettuce show a very wide range of values. In a pot experiment of Gelman (2014), practically no accumulation was detected in rooftop gardens in Helsinki. On the other hand, Jia et al. (2018) found that the total concentrations of 16 PAHs in samples collected from near industrial areas of Shanghai ranged between 132.0 and 319.2 μg/kg. Concentration of total PAHs in exposed lettuce plants in our study was 205.5, which falls into this range, indicating highly polluted conditions.

Strong correlation was established between BCF and molecular weight (Spearman’s rank correlation:* p* = 0.009, S = 1315.5, rho =  − 0.6121725). The lower molecular weight (LMW) PAH compounds were predominant after the treatment, which is in consistency with other studies (e.g. Lei et al. 2011; Wang et al. 2017; Jia et al. 2018). Naphthalene had the highest concentration (72 µg/kg), it is also reported to be one of the dominant PAHs in bioaccumulation studies (Busso et al. 2018). In general, highly lipophilic PAH molecules (heavy PAHs) show lower accumulative potential than the less lipophilic ones (light PAHs) (Paraíba et al. 2010).

The main conclusion can be that using the Guideline, accumulation studies can be carried out under laboratory conditions, using known and pre-set exposure and environmental concentration. Also, the Guideline allows a high level of variability (timing of treatments, comparison between different species, etc.). As the composition of the test material is known, accumulated materials can be clearly correlated with the composition of the sample.
